# Web-based objective and structured assessment of point-of-care lung ultrasound skills in resource-limited settings

**DOI:** 10.1186/s12909-024-05925-x

**Published:** 2024-08-28

**Authors:** Veronique Suttels, Ines Chichignoud, Prudence Ablo Wachinou, Jacques Daniel Du Toit, Pierre-André Mans, Juan Manuel Blanco, Gildas Agodokpessi, Thomas Brahier, Mary-Anne Hartley, Elena Garcia, Noémie Boillat-Blanco

**Affiliations:** 1 Department of Infectious diseases, Lausanne University Hospital and University of Lausanne, Lausanne, 1011 Switzerland; 2 National Teaching Hospital for Tuberculosis and Respiratory Diseases (CNHU-PPC), Cotonou, Benin; 3 MRCWits Rural Public Health and Health Transitions Research Unit (Agincourt), Faculty of Health Sciences, University of the Witwatersrand, Johannesburg, South Africa; 4 Department of Family Medicine, Cecilia Makiwane Hospital, East London, South Africa; 5 Department of Epidemiology and Health Systems, Centre for Primary Care and Public Health (Unisanté), University of Lausanne, 10 Route de La Corniche, 1010 Lausanne, Switzerland; 6 Intelligent Global Health Research Group, Swiss Institute of Technology (EPFL), 1015 Lausanne, Switzerland; 7 Emergency department, Lausanne University Hospital and University of Lausanne, 1011 Lausanne, Switzerland

**Keywords:** LUS-OSAUS, Lung POCUS, Low- and middle- income countries, Training, quality control

## Abstract

**Background:**

Objective assessment of skills after training is essential for safe implementation of lung point-of-care ultrasound (POCUS). In low-and middle-income countries (LMIC) there is a need for assessment tools without onsite experts to scale up POCUS access. Our objective is to develop a web-based assessment tool and evaluate trainees across different countries and at different time points after initial lung POCUS training.

**Methods:**

We adapted the objective and validated lung ultrasound score (LUS-OSAUS) to a web-based tool with quiz and practical skills test. Trainees were evaluated after a short (4-day) standardized lung POCUS training and were classified in distinct groups according to (i) their geographical location (Benin vs. South-Africa) and (ii) time elapsed since training (Benin 0 months vs. Benin 6 months). The Benin 6 months group had minimal continuous education. Skills test images were read by two blinded experts. We report the overall success rates and then compare these rates based on location and timing since training, using the Fischer’s exact test.

**Results:**

A total of 35 out of 43 participants completed the online LUS-OSAUS quiz and skills test. The overall success rate was 0.84 (95%CI 0.80–0.88), with lower success rates for “correct depth” 0.54 (0.37–0.71), “correct assessment of pleura” 0.63 (0.45–0.79) and “conclusion” 0.71 (0.54–0.85). There were no differences based on location, with respective rates of 0.86 (0.80–0.92) and 0.83 (0.75–0.91) (*p*-value = 0.125) for Benin and South Africa at 0 months, respectively. Similarly, there were no differences according to timing with success rates of 0.86 (0.80–0.92) and 0.82 (0.72–0.93) (*p*-value = 0.563) for Benin at 0 months and 6 months, respectively.

**Conclusion:**

Web-based objective and structured assessment of lung POCUS skills in LMIC following a short-standardized training is feasible and has a good overall success rate with consistent results across regions and up to 6 months after training given minimal continuous education. Overall, technical and POCUS-based clinical conclusion skills are the most difficult to acquire.

**Supplementary Information:**

The online version contains supplementary material available at 10.1186/s12909-024-05925-x.

## Background

Point-of-care ultrasonography (POCUS) of the lungs is increasingly used as a first-line imaging tool to manage patients with respiratory symptoms [1]. With the advent of portable and affordable devices, POCUS has become particularly attractive to low- and middle-income countries (LMICs), as these tools overcome typical imaging barriers such as the absence of radiology services or high patient fee [2–5].

However, insufficiently or inadequately trained physicians might harm patients through inaccurate diagnoses or inappropriate use of POCUS. Recently, POCUS was labeled as a potential major health technology hazard by the Joint Commission on Accreditation of Healthcare Organizations and the Emergency Care Research Institute, emphasizing the risks of adopting the tool without essential precautions such as validated training [6, 7]. For each subspecialty, including lung POCUS, ensuring adequate training programs and standardized skills assessment are key to develop its safe and effective use. While training efforts are progressing in high-income countries, with at least 35% of US medical schools integrating POCUS in their curriculum [8], LMICs often lack formal training, onsite experts, and early exposure in undergraduate and postgraduate curricula [9, 10]. Web-based solutions are already readily used in emergency medicine residency programs and can be interesting in LMIC to overcome the initial need of local experts [11]. The significant heterogeneity among existing lung POCUS courses, along with deficits in skill evaluation, necessitates research involving theoretical and practical assessments after training. To address this gap, a lung ultrasound objective structured assessment of technical skills (LUS-OSAUS) score was developed in 2016 [12] and validated in 2020 for medical undergraduate students in Europe [13]. Our objective is (i) to adapt the LUS-OSAUS score for web-based application in resource-limited settings and (ii) evaluate trainees across various countries and at two distinct time points (immediately after initial training and after a 6-month period) after a standardized short lung POCUS training.

## Methods

### Web-based LUS-OSAUS tool development adapted to LMICs

The LUS-OSAUS score was adapted to an online quiz tool and practical skills test with content adjusted to align with LMIC epidemiology. The LUS-OSAUS score evaluates the trainee’s expertise according to 6 areas (test indication, systematic lung ultrasound examination, technical skills, interpretation of lung ultrasound findings, documentation and conclusion), which were strictly maintained. Accross these 6 areas, 17 items are included [12]. The original items were slightly modified to make the quiz questions more fluent. Our adapted LUS-OSAUS tool has 6 areas and 18 items (summarized in Table 1, overview of minor modifications to the original score are available in appendix Table 1).

**Fig. 1:**
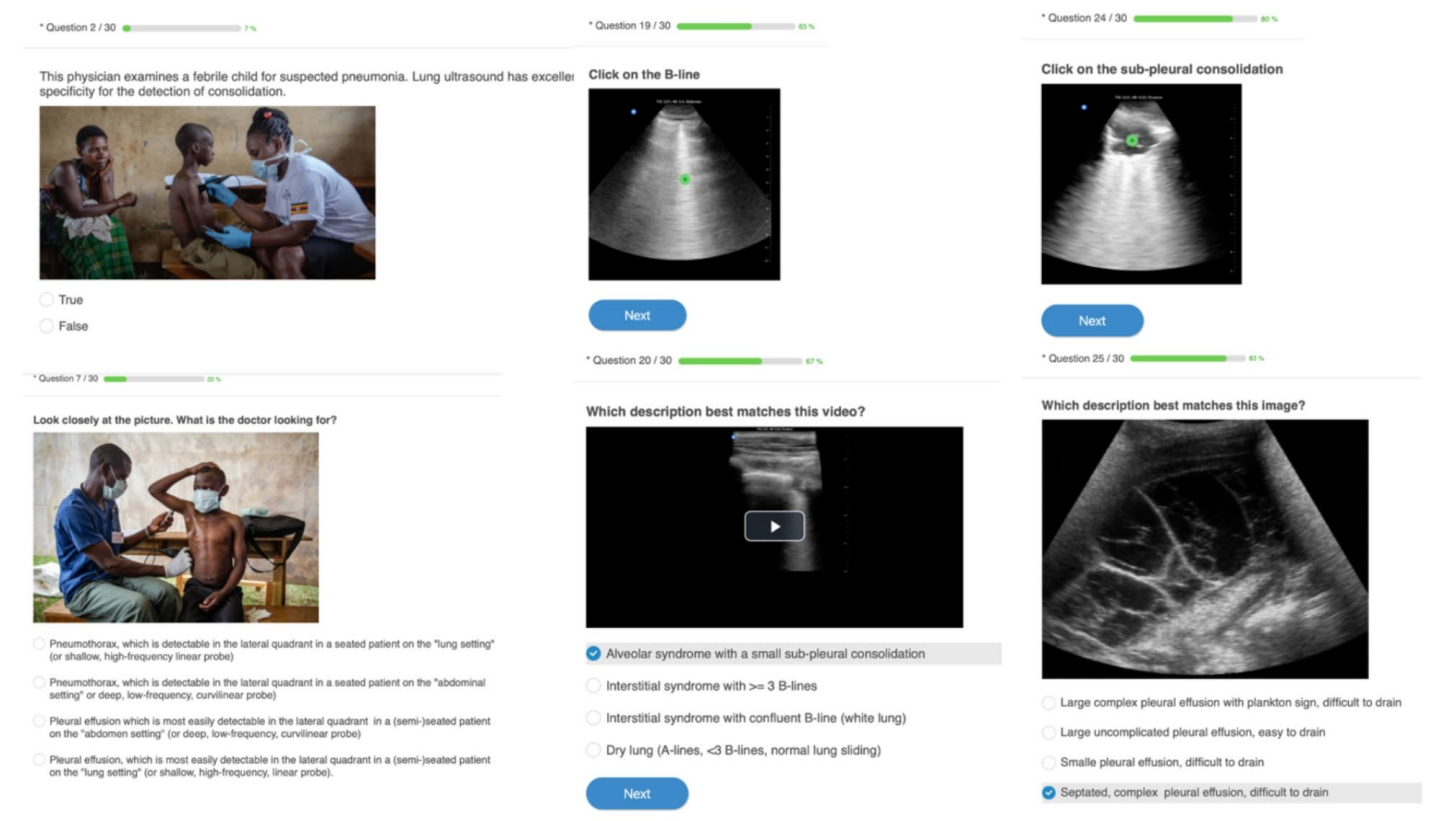
Example of online quiz questions adapted to a low-resource setting and based on the LUS-OSAUS score (available in a French and English version)

To assess these 18 items, we developed a 30-questions online quiz (Fig. 1, full quiz available in appendix Table 2) and 5 practical challenges (appendix Table 3). The 5 practical challenges consisted of capturing and uploading 5 s videos of the curtain sign in the right and left lateral quadrants, anterior lung sliding in M-mode, a 5 cm depth view of the pleural line and a posterior view of the pleural-line. Each challenge was evaluated for preset (or choice of probe), depth, gain, interpretability of the image, saving and labelling. The practical challenges were scored by 2 independent and certified remote LUS readers. A third reader was asked to grade in case of disagreement between the first two readers. All readers were blinded for the participant’s characteristics.

Appendix table 4 shows the link between the evaluated items, the online quiz questions and practical challenges. The original score evaluated every item on a scale of 1 to 5. With the adapted score, every question of the quiz was rated as correct (1 point) or not (0 point) and each item was evaluated through 1 to 5 questions. Every practical challenge (5 challenges) allowed to assess 6 items and were evaluated as passed (1 point per item and per challenge) or not (0 point). Participants could earn a maximum of 30 points for correctly answering online quiz questions and 30 points for successfully completing practical challenges. This added up to a maximum of 60 points achievable per participant. Since each item is evaluated through 1 to 5 questions, we defined an item as acquired based on a minimal percentage of correct answers per item: (n-1)/n (where n represents the number of questions for a single item) [14]. Levels of success were categorized as follows: good if the proportion of trainees who succeeded was ≥ 0.80, moderate if the proportion ranged from 0.70 to 0.79 and low if it was < 0.70.

**Table 1:** Adapted LUS-OSAUS score with 6 areas and 18 items

Area (*n* = 6)			Item (*n* = 18)
Indication			
			Indication: evaluates the indication for lung ultrasound, suggests focused questions
Systematic lung ultrasound examination			
			Performs lung ultrasound systematically
			Performs lung ultrasound on the basis of a focused question and places patient accordingly
Technical skills			
			Correct choice of transducer/preset
			Correct depth
			Correct gain
			Saves images correctly
			Labels anatomical position correctly
			Interpretability of the images
Findings			
			Correct assessment of pleura
			Correct assessment of B-lines
			Correct assessment of consolidations
			Correct assessment of pleural effusion
			Correct assessment of diaphragm
			Correct assessment of M-mode
			Correct assessment of whether ultrasound-guided thoracentesis is safe
Documentation			
			Documents findings in patient’s chart
Conclusion			
			Able to make a diagnosis on the basis of lung ultrasound findings and able to integrate lung ultrasound findings with patient’s history

### Material used

The online quiz questions were developed using a platform to create interactive learning assessments (ProProfs^®^). For the practical challenges, participants were provided with an ultrasound on a chip POCUS device (Butterfly IQ ^®^). Test videos were recorded and uploaded on a secured cloud system and anonymized for the grading readers.

### Standardized training content and trainee groups

The initial training was the same for all participants and consisted of a 4-hour lung ultrasound theory session, a 2-hour small group (max 4 participants) training on a healthy volunteer, a 6-hour small group (max 4 participants) on hospitalized patients and a 2-hour recapulative session, spread over 4 days (appendix Fig. 1). Trainers were Swiss LUS experts. Trainees were classified in three distinct groups based on their geographical location and the timing since their initial training. The first group (Benin 0 months) consisted of 18 West-African health care professionals trained at the National University Hospital for Tuberculosis and Lung Diseases in Cotonou, Benin in February 2022 and was evaluated immediately after training. The second group (South-Africa 0 months) consisted of 17 South-African physicians trained at the district-level public hospital of Tintswalo, Acornhoek, Mpumalanga Province in April 2022 and was also evaluated immediately after training. The third group (Benin 6 months) consisted of 8 physicians working in the National Teaching Hospital for Tuberculosis and Lung Diseases in Cotonou, Benin and were trained in September 2021. Since their initial training, this group had weekly 1-hour voluntary POCUS case meetings and on-demand POCUS supervision for selected cases. Participants were evaluated six months after their initial training, in March 2022.

### Statistical evaluation

We report the overall success rates of all participants (percentage of points obtained out of a total of 60 points), as well as success rates by LUS-OSAUS area (*N* = 6) and by individual items (*N* = 18).

To assess differences by region and by time elapsed since training, we analyze and compare the success rates overall, by area and by item between Benin 0 months and South Africa 0 months as well as between Benin 0 months and Benin 6 months, respectively, using the Fischer’s exact test. All statistical analyses are performed using the STATA statistics software package, version 17.0.

## Results

A total of 35 out of 43 trainees completed and uploaded the LUS-OSAUS quiz and skills test and are part of the analysis. No participant was previously certified in ultrasonography. However, the groups showed some differences in terms of previous lung POCUS practice as summarized in Table 2. In the Benin 0 months group, 5 (31%) already practiced unsupervised lung ultrasound before the skills assessment (3 practiced daily and 2 on a weekly basis in the last 6 months) without previous standardized training. In the South Africa group, all participants were novices. In the Benin 6-month group, 6 trainees (86%) had daily unsupervised practice of lung ultrasound during the time between training and assessment.

**Table 2:** Summary of group characteristics. * with quiz and skills completed and uploaded

	Group 1	Group 2	Group 3
	Benin 0 months	South Africa 0 months	Benin 6 months
Date of training	February 2022	April 2022	September 2021
Number of participants	18	17	8
Number of participants included in the analysis*	16	12	7
Previous lung POCUS practice			
Daily	3	0	6
Weekly	2	0	0
Monthly	0	0	0
Never	11	12	1

The overall success rate was 0.84 (95%CI 0.80–0.88). The two areas that exhibited the highest levels of success (≥ 0.80) are “indication of lung POCUS”, with a score of 1 (95% CI 0.90-1), and “documentation of findings”, with a score of 0.91 (95% CI 0.77–0.98). The areas with a moderate level of success (0.70–0.79) are “findings on lung POCUS”, with a score of 0.77 (95% CI 0.60–0.90) and “conclusion”, with a score of 0.71 (95% CI 0.54–0.85). The area with the lowest level of success (< 0.70) is “technical skills” with a score of 0.66 (95% CI 0.48–0.81). Among the 18 items, a majority (14/18, 78%) exhibited the highest levels of success (≥ 0.80), with maximum scores achieved by assessment of pleural effusion, B-lines, and indication of lung POCUS. Two items had a moderate level of success (0.70–0.79): “correct choice of transducer/preset” with a score of 0.71 (95% CI 0.54–0.85) and “conclusion” also with a score of 0.71 (95% CI 0.54–0.85). Two items had the lowest level of success (< 0.70): “correct depth” with a score of 0.54 (0.37–0.71) and “correct assessment of pleura” with a score of 0.63 (0.45–0.79). Although the depth choice was often incorrect, there was a high level of success on “interpretability of the images” at 0.80 (95%CI 0.63–0.92).

**Fig. 2:**
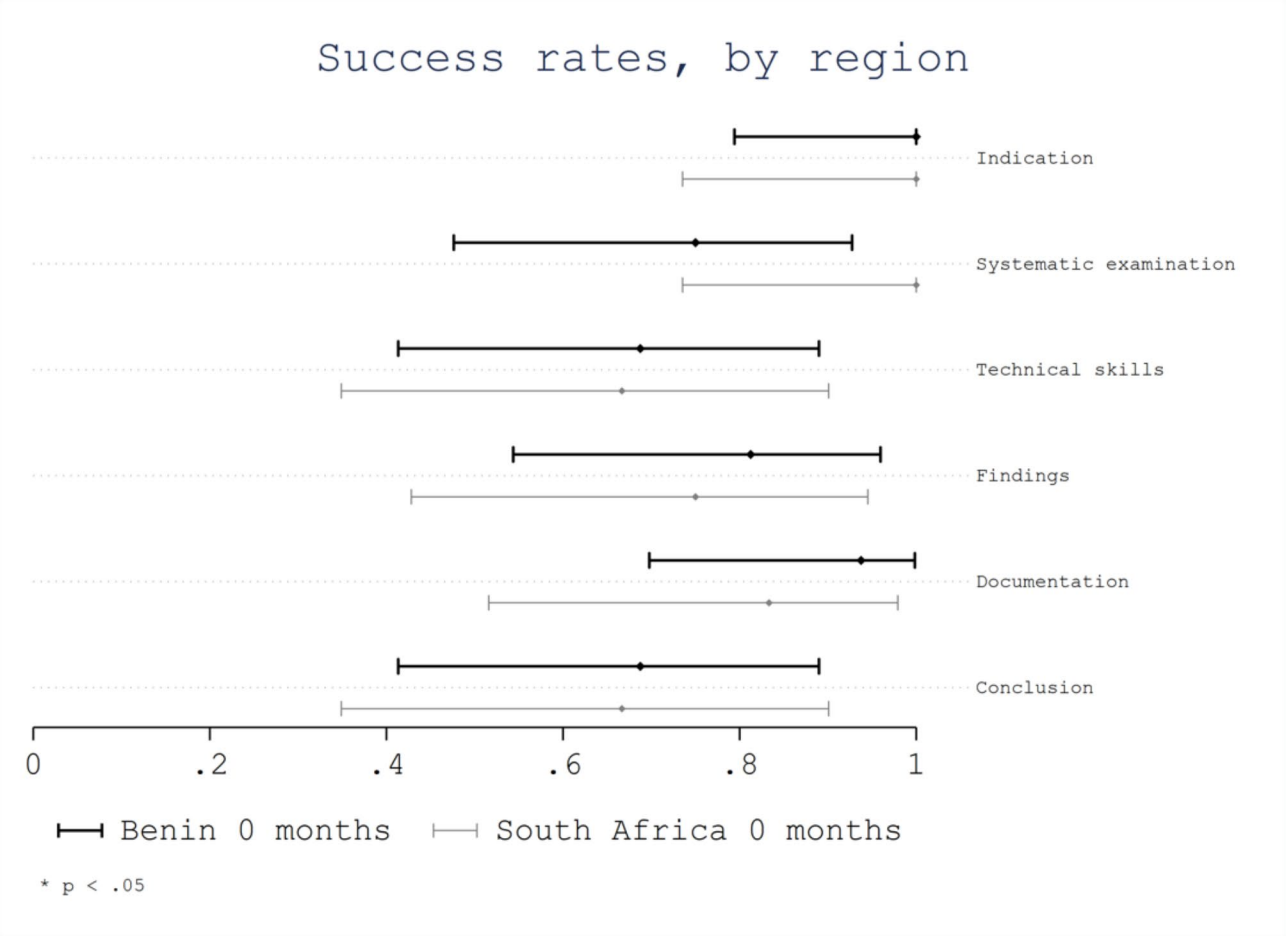
Success rate per area by region

**Fig. 3:**
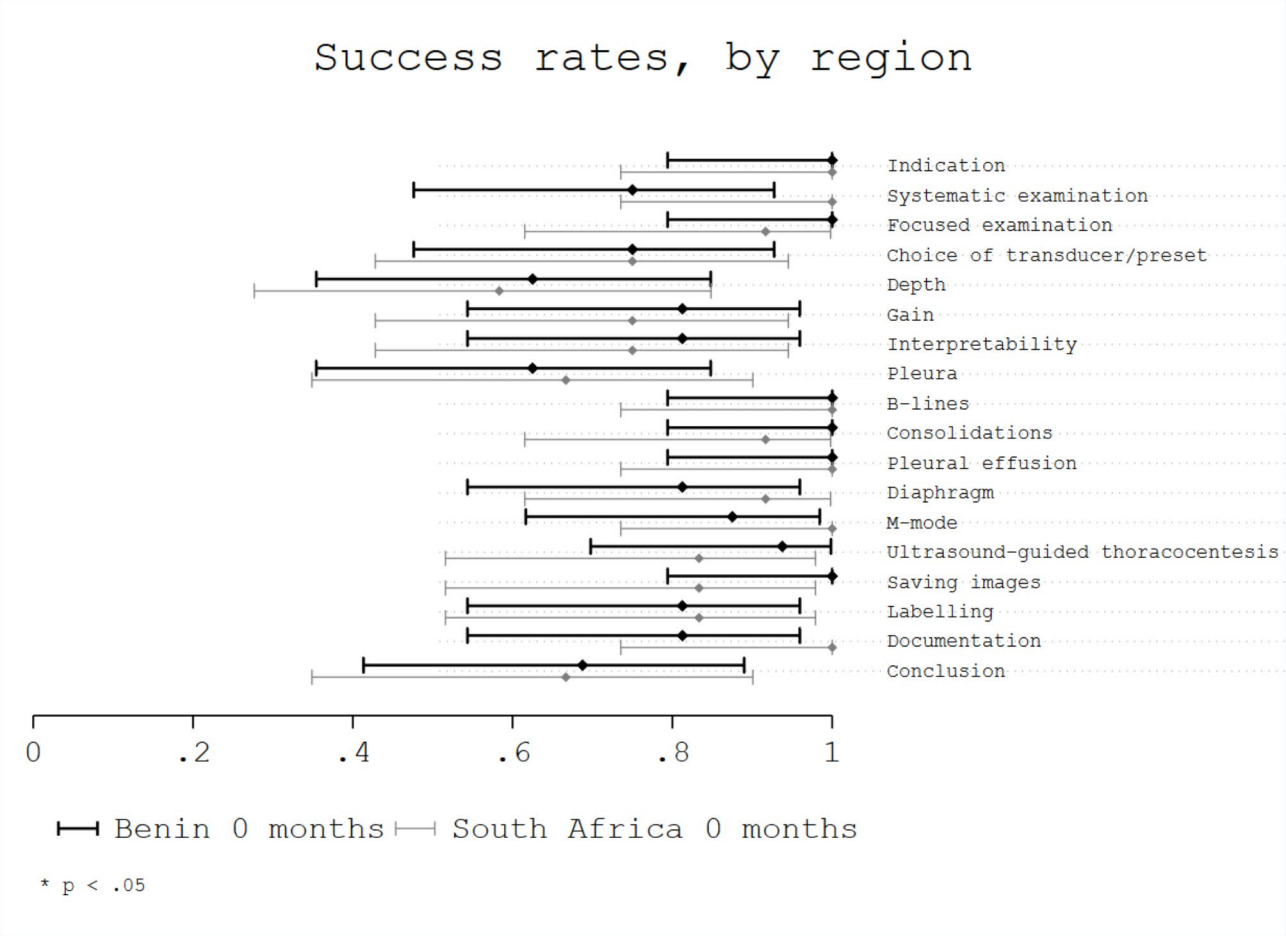
Success rate per item by region

Considering geographical region (Benin 0 months vs. South Africa 0 months), no significant differences were observed, with respective overall success rates of 0.86 (95%CI 0.80–0.92) for Benin 0 months and 0.83 (95%CI 0.75–0.91) for South Africa 0 months (*p* = 0.13). No significant differences were observed in the success rates of evaluated areas based on the region (Fig. 2). However, trainees from Benin showed a non-significant lower success rate for the area “systematic examination” with a score of 0.75 (95% CI 0.48–0.93) compared with trainees from South Africa with a score of 1.0 (95% CI 0.74-1.0; *p* = 0.11). No significant differences were observed in the success rates of evaluated items based on the region (Fig. 3).

**Fig. 4:**
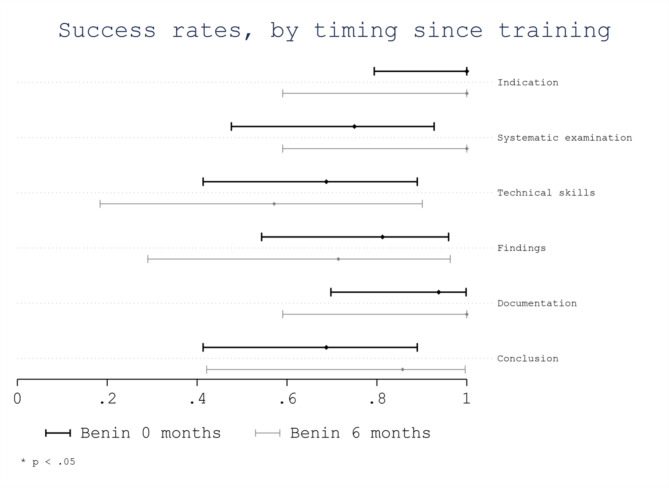
Success rate per area by timing elapsed since training

**Fig. 5:**
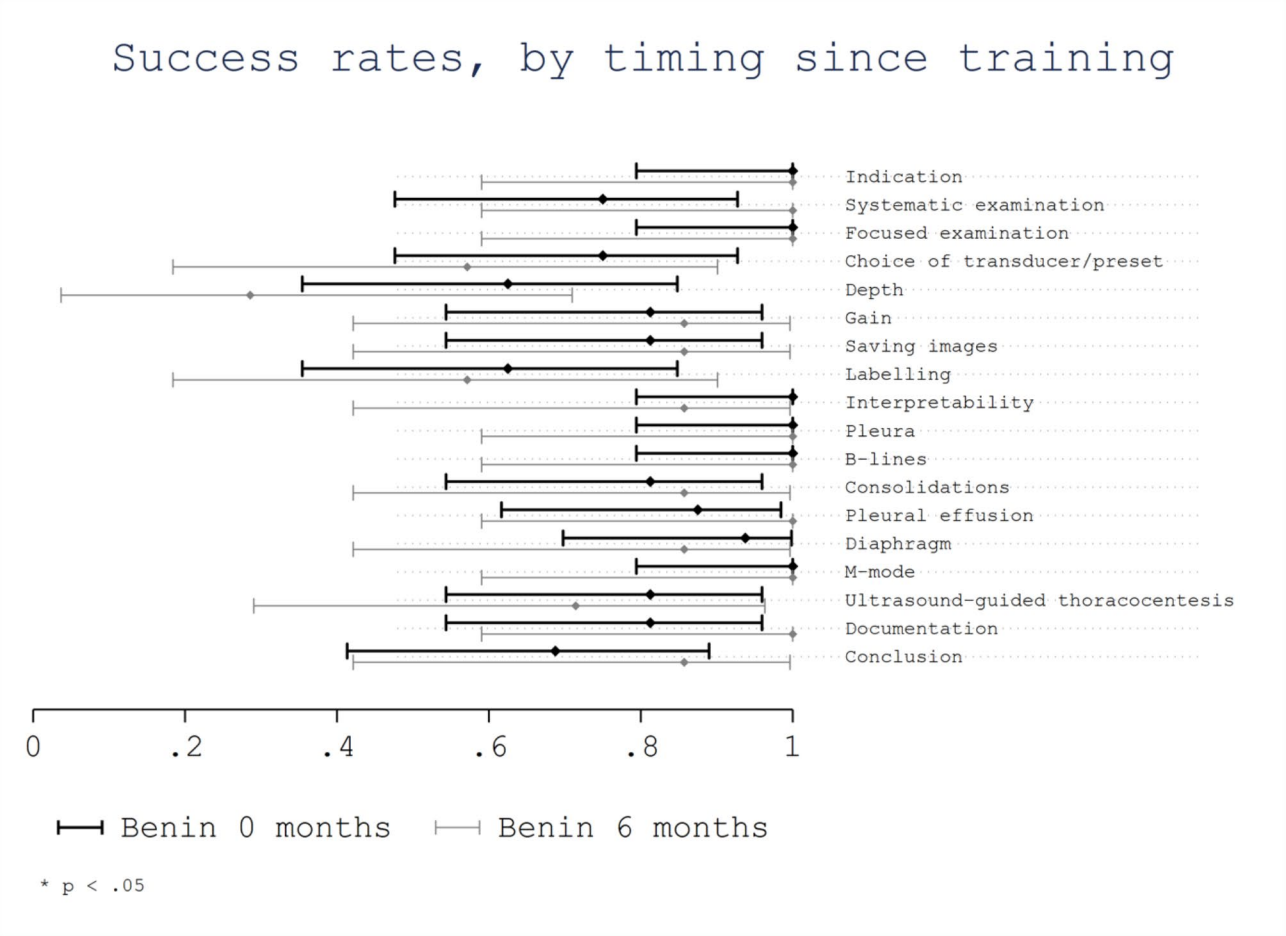
Success rate per item by timing elapsed since training

Considering time elapsed since training (Benin 0 months vs. Benin 6 months) no significant differences were observed. The overall success rate was 0.86 (95%CI 0.80–0.92) for Benin 0 months and 0.82 (95%CI 0.72–0.93) for Benin 6 months (*p* = 0.56). No significant differences were observed in the success rates of evaluated areas (Fig. 4). However, trainees from Benin 0 months showed a non-significant lower success rate for the area “systematic examination” with a score of 0.75 (95% CI 0.48–0.93) compared with trainees from Benin 6 months with a score of 1.0 (95% CI 0.59-1.0; *p* = 0.27). In addition, trainees from Benin 0 months showed a non-significant lower success rate for the area “conclusion” with a score of 0.69 (95% CI 0.41–0.89) compared with trainees from Benin 6 months with a score of 0.86 (95% CI 0.42-1.0; *p* = 0.62). No significant differences were observed in the success rates of evaluated items based on the time since training, although trainees from Benin 0 months showed a non-significant higher success rate for some scanning technical skills as the “correct choice of transducer/preset”, the “correct depth” and the “labelling of anatomical position” (Fig. 5).

## Discussion

Web-based objective and structured assessment of theoretical and practical lung POCUS skills in low-resource settings is feasible and revealed a good overall success rate of 0.84 (95%CI 0.80–0.88) after a short 4-day training. In line with our findings, previous studies on learning curves of various POCUS domains showed that lung POCUS is indeed a relatively easy skill to acquire [15, 16]. To reach basic technical competence, it is estimated that 5 to 25 supervised lung POCUS exams are necessary [16–19]. The following items appear the most challenging: (1) setting the correct depth, (2) correct assessment of the normal pleura and (3) POCUS based clinical conclusions.

First, it is important to note that even within a context of longitudinal (e.g., 6 months) lung POCUS learning programs correct depth and axis of the probe remain challenging [15, 16]. Depth is particularly important in assessing the lower lung quadrants allowing to correctly visualize the diaphragm to make a clear distinction between thorax and abdomen. A study after short (4.5 h) training of respiratory therapists, showed that the lower lung quadrants are more challenging to evaluate (98.4 ± 1.8% of upper lung zone images were interpretable versus 91.3 ± 9.5% of lower lung zone images were interpretable) [20].

Second, our study also points out that trainees have particular difficulties with recognizing normal pleura as opposed to the facility to recognize B-lines and pleural effusions. After a 4-day training on the BLUE-protocol in Italy, internal medicine residents as compared to experienced sonographers performed “excellent” for the recognition of B-lines and pleural effusion ((with kappas of 0.90 (0.88–0.92) and 0.82 (0.81–0.83) respectively). However, similar to our findings, recognition of the normal lung was more challenging with a kappa of 0.65 (0.61–0.69) [21]. A recent study showed that after a very short 1.5 h training for ICU nurses, the normal pleura and lung sliding are also significantly more challenging to recognize as compared to the B-line pattern (0.34 (0.24–0.48) correct interpretation versus 0.88 (0.76–0.92) respectively) [22]. Our participants tend to err on the side of seeing pathology. For instance, Z-lines which are small subpleural artefacts seen in 80% of the normal population [23], are mistakenly taken for subpleural consolidations (overinterpretation). In specific settings where subtle pleural lesions are important for diagnosis (e.g., diagnosis of pulmonary tuberculosis), 50.8% of inter reader mismatches are between normal pleura and subpleural lesions of less than 1 cm [24]. Training in this setting is reportedly more time-intensive requiring on-site presence of a skilled trainer over a longer period of time [25].

Third, trainees had difficulties integrating POCUS findings into clinical-decision making. This skill tends to improve with practice as shown by a tendency to better performance 6 months after training. This is consistent with a study in Rwanda where learners had better thoracic POCUS integration skills 58-weeks post training. However, in this study, training course was intensive with frequent follow-up training [26] A study in Ghana using clinical vignettes to assess the skill of integrating cardiopulmonary POCUS findings found a stable performance 9–11 months after a short initial training. This might be because of a focus on cardiac and abdominal POCUS (83% and 92% of participants with weekly practice respectively) as compared to lung POCUS (78% weekly practice) [27].

Regarding timing after initial training, success rates remained high after 6 months including minimal continuous education as described above. The overall consistency of POCUS skills after short (1.5 h to 4 day) training courses over time aligns with previous research in low- [25, 27] as well as high-income countries [20, 28, 29].

To improve lung POCUS skills over time, additional continuous ultrasound training including direct supervision and targeted ultrasound rounds lead to significantly better results [29–31]. However, current limited opportunities in LMIC for supervised practice have been identified as an important barrier [32, 33]. This barrier might be partly overcome with remote supervision applications and artificial intelligence (AI) support [25, 34]. We further suggest that besides technical skills, continuous training should also give particular attention to integrating POCUS findings into clinical decision-making.

No significant difference in success rate was found based on geographic region (South-Africa vs. Benin). Globally, online lung POCUS training has gained substantial momentum in recent years [28, 35]. These consistent outcomes across regions are encouraging for the potential and broader applicability of this web-based evaluation tool for LMICs.

Finally, collaborative efforts involving the academic and the private sector, governments and NGOs are crucial to establish longitudinal training programs where needed, ensure quality control including AI support and promote safe and sustainable adoption of lung POCUS in LMIC [25, 36, 37].

### Limitations

The participants in our study might not accurately represent all healthcare professionals learning in low-income practice environments. Those who subscribed for training expressed a personal interest in gaining ultrasound skills and sometimes traveled far for this training. As a result, the performance of this specific group might not be applicable to a wider range of healthcare professionals. Additionally, limitations include the small number of participants involved in the study, their heterogeneity (some with previous experience) and difficulties with connectivity or informatics (8 out of 43 trainees could not fully upload their test). The small number of participants in each group (based on geographical regions and timing since training) provide limited power for comparison. Another limitation is linked to the group definition” timing after training”. Indeed, we did not follow up longitudinally the same group of trainees, but compared two different groups of trainees according to the timing elapsed since training.

## Conclusion

Web-based objective and structured assessment of lung POCUS skills in LMIC following a short standardized training is feasible and showed a good overall success rate among trainees with consistent results across regions and up to 6 months after training given minimal continuous education. Overinterpretation is an important beginner’s mistake and the acquisition of technical and integrative clinical skills can be challenging. Continuous training modules can improve technical skills and should also give particular attention to the integration of POCUS findings in clinical-decision making. Collaborative efforts are needed for further standardization of lung POCUS training curricula and validation in LMIC.

### Electronic supplementary material

Below is the link to the electronic supplementary material.

Supplementary Material 1

## Data Availability

The links to course content and online quiz are available in the appendix. The datasets used and/or analysed during the current study are available from the corresponding author on reasonable request.

## Abbreviations

AI: Artificial intelligence
ICU: Intensive care unit
LMIC: Low- and middle-income countries
LUS: Lung ultrasound
LUS-OSAUS: Lung ultrasound objective and structured assessment ultrasound score
NGO: Non-governmental organization
POCUS: Point-of-care-ultrasound
USA: United States of America
