# Supplementary material for: Web-based objective and structured assessment of point-of-care lung ultrasound skills in resource-limited settings
Source: BMC Med Educ. 2024 Aug 28;24:939. doi: 10.1186/s12909-024-05925-x (PMC11360711; doi:10.1186/s12909-024-05925-x)

**APPENDIX**

Table 1: Modifications of the LUS-OSAUS score to an adapted score for LMICs.

|  | Original LUS-OSAUS score | Adapted score for LMICs |  |
| --- | --- | --- | --- |
| Area | Item (n=17) | Item (n=18) | Comments |
| Indication |  |  |  |
|  | Evaluates the indication for lung ultrasound | Idem | we have grouped these two items into one: “Indication” |
|  | Suggests focused questions that can be examined by lung ultrasound |  |  |
| Systematic lung ultrasound examination |  |  |  |
|  | Performs lung ultrasound systematically | Idem: “Systematic examination ” |  |
|  | Performs lung ultrasound on the basis of focused question | Performs lung ultrasound on the basis of focused question and places patient accordingly | we grouped the item "Performs lung ultrasound on the basis of focused question" and "correct placement of patient" as we could not directly observe the exam (remote tool): “Focused examination” |
| Technical skills |  |  |  |
|  | Correct placement of patient |  | grouped with item " Performs lung ultrasound on the basis of focused question” in the area “Systematic lung ultasound examination”: “Focused examination” |
|  | Correct handling of the transducer |  | this item is not evaluated |
|  | Correct choice of transducer/preset | Idem |  |
|  | Correct depth | Idem |  |
|  | Correct gain | Idem |  |
|  |  | Saves images correctly | added |
|  |  | Labels antomical position correctly | added |
|  |  | Interpretability of the images | added |
| Findigs |  |  |  |
|  | Correct assessment of pleura | Idem |  |
|  | Correct assessment of B-line | Idem |  |
|  | Correct assessment of consolidations | Idem |  |
|  | Correct assessment of pleural effusion | Idem |  |
|  |  | Correct assessment of diaphragm | added |
|  |  | Correct assessment of M-mode | added |
|  | Correct assessment of whether ultrasoundguided thoracentesis is safe | Idem |  |
| Documentation |  |  |  |
|  | Documents findings in patient’s chart | Idem |  |
| Conclusion |  |  |  |
|  | Able to make diagnosis on the basis of lung ultrasound findings | Idem | we have grouped these two items into one: “Conclusion” |
|  | able to integrate lung ultrasound findings with patient’s history |  |  |

Table 2: Complete electronic quiz questions adapted to a LMIC setting and based on the LUS-OSAUS score (available in a French and English version).

Link to English online quiz: <https://www.proprofs.com/quiz-school/ugc/story.php?title=assessment-of-lung-ultrasound-skillszo>

Link to French online quiz: <https://www.proprofs.com/quiz-school/ugc/story.php?title=evaluation-des-comptences-en-ultrason-pulmonaire>

Link to skills test (bilingual): <https://www.proprofs.com/quiz-school/ugc/story.php?title=pratique_5ig>

| Questions | Image or video | Multiple choice |
| --- | --- | --- |
| ****Q1.** This physician is examining a patient who presents with acute chest pain and dyspnea. Which statement is true?** | 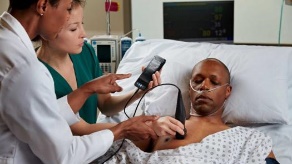 | A.Chest pain and dyspnea is only an indication to perform a lung ultrasound if the chest X-ray was non-conclusive  B. Chest pain and dyspnea is always a good indication to perform a lung ultrasound, here the doctor is looking for an anterior pneumothorax **(correct)**  C. Chest pain and dyspnea is only an indication to perform a lung ultrasound if the patient is also febrile. The physician is looking for pneumonia  D. Chest pain and dyspnea is always a good indication to perform a lung ultrasound. Here the doctor is looking for a pleural effusion |
| Q2. This physician examines a febrile child for suspected pneumonia. Lung ultrasound has excellent sensitivity and specificity for the detection of consolidation. | 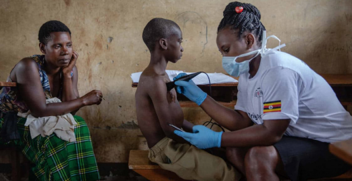 | A.True **(correct)**  B.False |
| Q3. **You suspect pulmonary embolism in a symptomatic high-risk patient. Which statement is true?** | 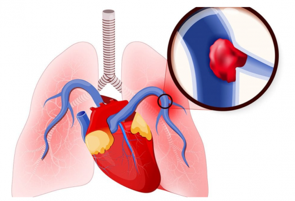 | A.Lung ultrasound allows direct visualization of thrombosis in the pulmonary veins. A normal lung ultrasound can rule out pulmonary embolism  B. Lung ultrasound does not directly visualize pulmonary embolism and can be normal. However, finding a DVT by ultrasound can rule in a pulmonary embolism **(correct)** |
| Q4. **In general, it is difficult to detect pleural effusion with lung ultrasound. Chest X-ray is a better option if available?** | 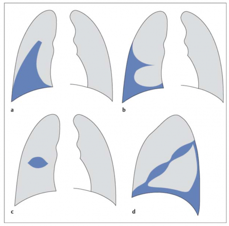 | A.True  B.False (**correct)** |
| Q5. **B-lines on lung ultrasound (indicating interstitial syndrome) has a very broad differential diagnosis (heart failure, interstitial lung disease, atypical pneumonia...) ?** | 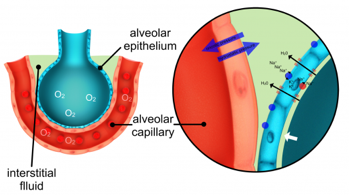 | A.True **(correct)**  B.False |
| Q6. **When doing a lung ultrasound for suspected pulmonary tuberculosis, which quadrants should you scan?** | 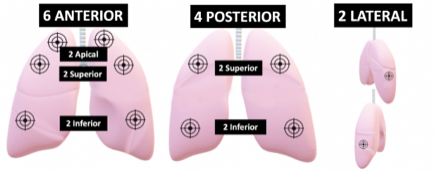 | A.Apical quadrant  B.All quadrant **(correct)**  C.Apical and lateral quadrants  D.Posterior superior quadrants |
| Q7. **Look closely at the picture. What is the doctor looking for?** | 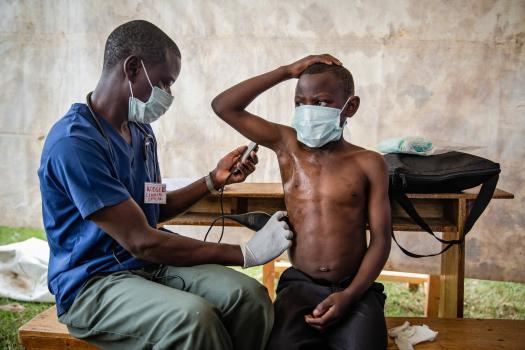 | A. Pneumothorax, which is detectable in the lateral quadrant in a seated patient on the "lung setting" (or shallow, high-frequency linear probe)  B. Pneumothorax, which is detectable in the lateral quadrant in a seated patient on the "abdominal setting" or deep, low-frequency, curvilinear probe)  C. Pleural effusion which is most easily detectable in the lateral quadrant in a (semi-)seated patient on the "abdomen setting" (or deep, low-frequency, curvilinear probe) **(correct)**  D. Pleural effusion, which is most easily detectable in the lateral quadrant in a (semi-)seated patient on the "lung setting" (or shallow, high-frequency, linear probe). |
| Q8. **Which description best matches this video?** | 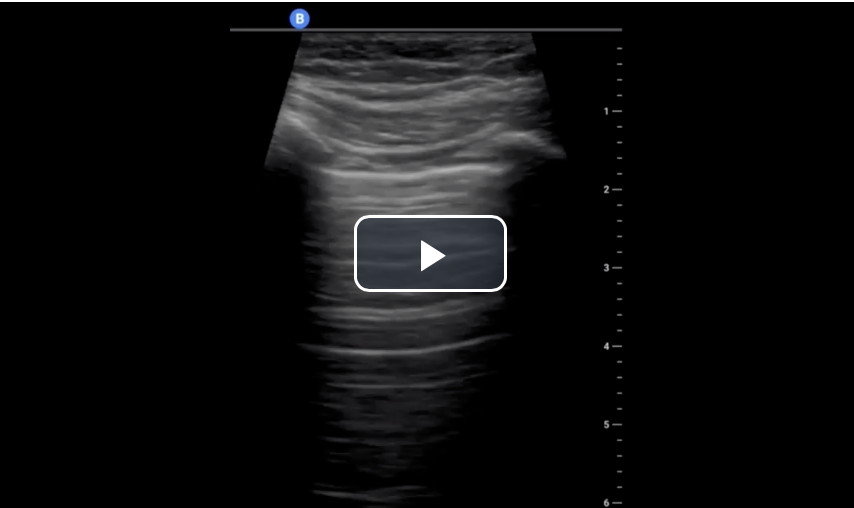 | A. Dry lung (A lines, normal lung sliding) **(correct)**  B. Pleural effusion  C. Interstitial syndrome (>= 3 B-lines per field)  D. Alveolar syndrome with a consolidation of >1cm |
| Q9. **Which description best matches this video?** | 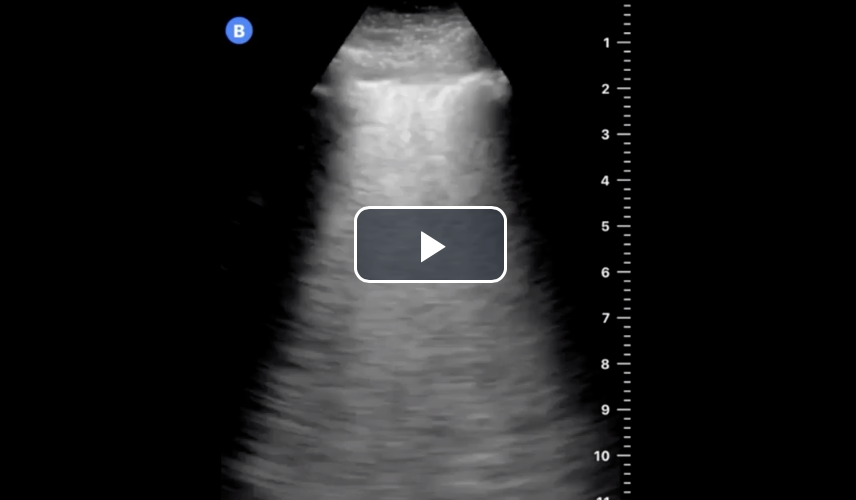 | A.Dry lung (A-lines, normal lung sliding)  B.Alveolar syndrome with major consolidation  C. Large pleural effusion (>1cm)  D. Irregular pleural line generating B-lines  **(correct)** |
| Q10. **Which description best matches this M-mode image?** | 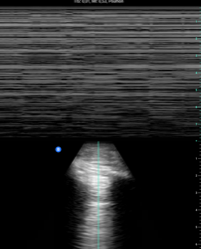 | A.Barcode sign (absence of lung sliding, as seen e.g. in pneumothorax) **(correct)**  B. Seashore sign (presence of lung sliding) |
| Q11. **Click on the pleural line** | 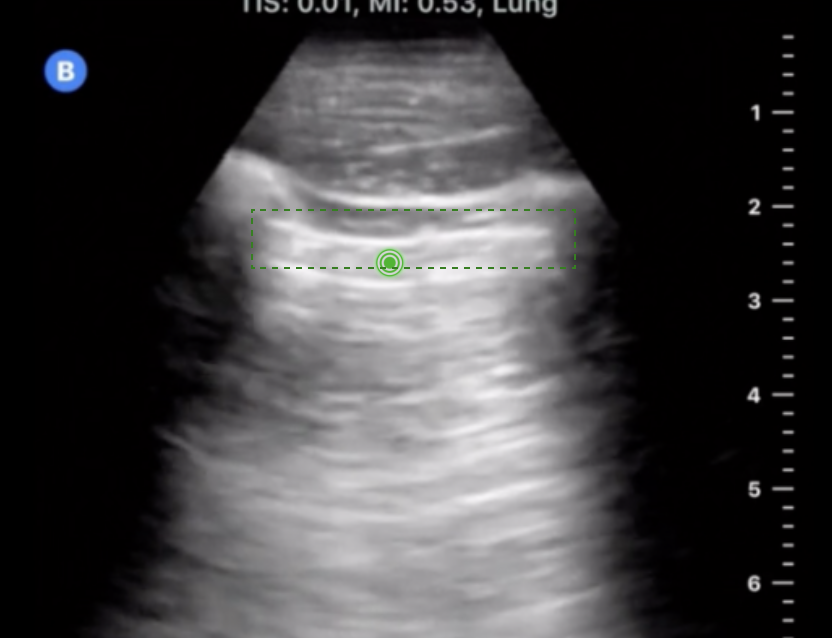 |  |
| Q12. **Which description best matches this video?** | 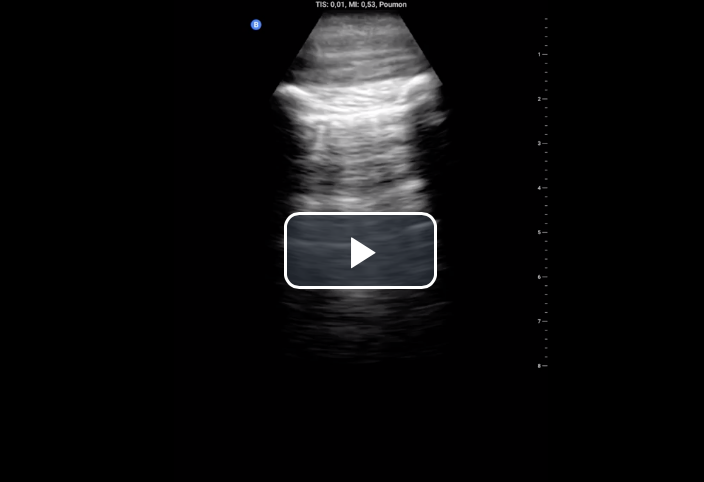 | A.Pneumothorax (absence of lung sliding)  B.Alveolar syndrome with a consolidation >1cm  C. Interstitial syndrome (>3 B-lines seen as comet-tail artefacts from the pleural line to the bottom of the image)  D. Dry lung (A-lines, normal lung sliding, <3 B-lines) **(correct)** |
| Q13. **Which description best matches this video?** | 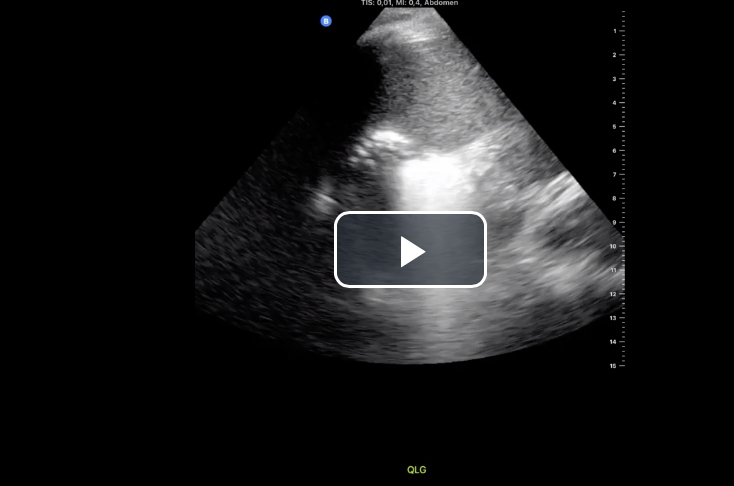 | A. Alveolar syndrome with a major consolidation ("tissue-like" sign)  B. Left pleural effusion  C. Curtain sign: the lung passes over the spleen, visualized in an "abdomen" pre-set **(correct)**  D. Interstitial syndrome (>= 3 B-lines) |
| Q14. **Which description best matches this video?** | 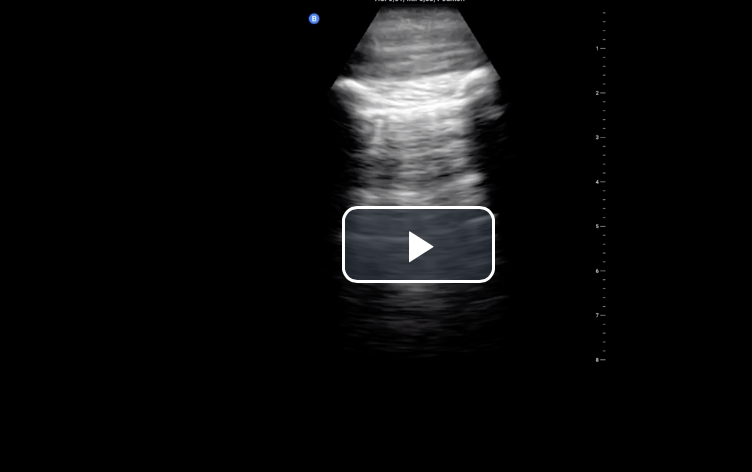 | A.Seahore signe (normal lung sliding) **(correct)**  B.Barcode sign (absent lung sliding) |
| Q15. **Which description best matches this video?** | 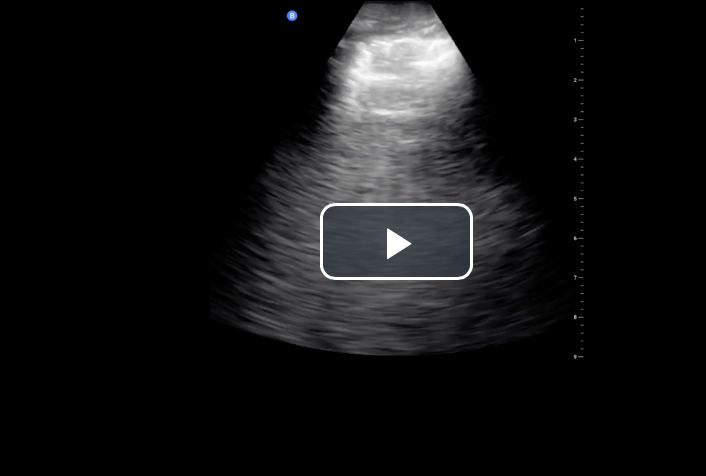 | A. Pneumothorax (absent lung sliding, lung-point visualized)  B. Irregular pleural line and small consolidation (<1cm  C. Dry lung (A-lines, <3 B-lines, normal lung sliding)  D. Pleural effusion |
| Q16. **Image of the right lateral quadrant in an "abdomen" pre-set. We observe the liver, the diaphragm and a large pleural effusion. Note the thoracic spine sign. Click on the pleural effusion.** | 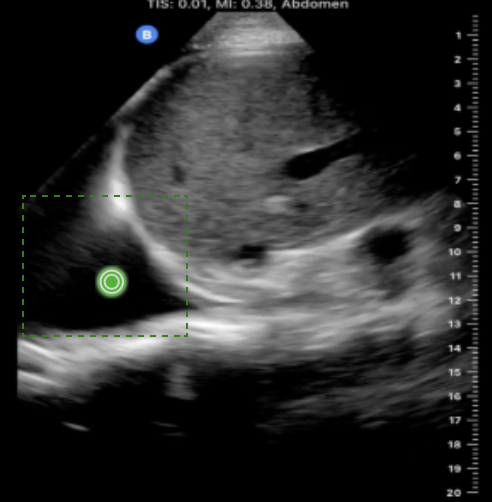 |  |
| Q17. **Which description best matches this video?** | 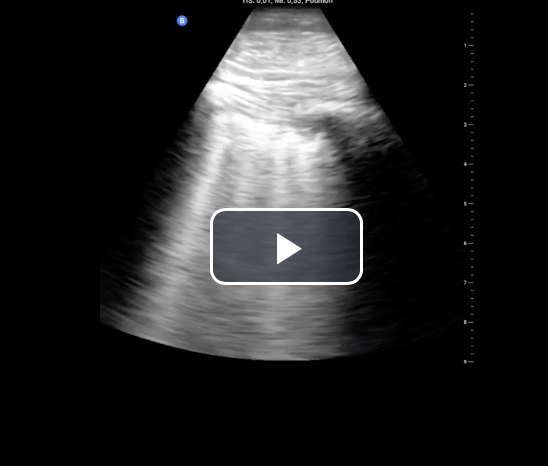 | A. Dry lung (A-lines, <3 B-lines, normal lung sliding)  B. Pneumothorax (absent lung sliding, lung-point visualized)  C. Alveolar syndrome with major consolidation ("hepatization")  D. Interstitial syndrome with >= 3 B-lines and a thickened pleural line **(correct)** |
| Q18. **Which description best matches this video?** | 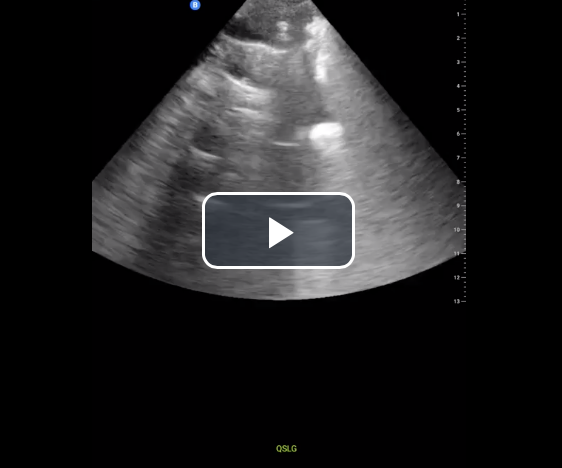 | A. Alveolar syndrome with major consolidation **(correct)**  B. Interstitial syndrome with >= 3 B-lines  C. Dry lung (A-lines, <3 B-lines, normal lung sliding)  D. Pneumothorax (absent lung sliding) |
| Q19. **Click on the B-line** | 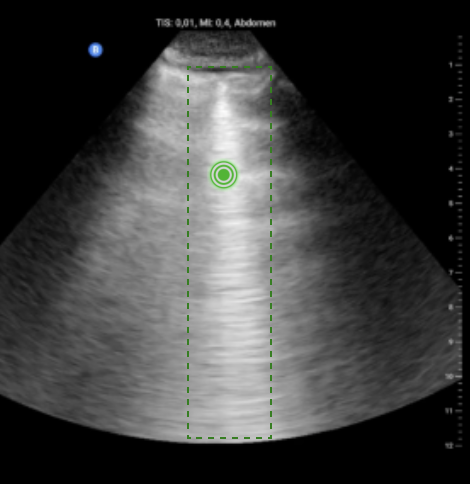 |  |
| Q20. **Which description best matches this video?** | 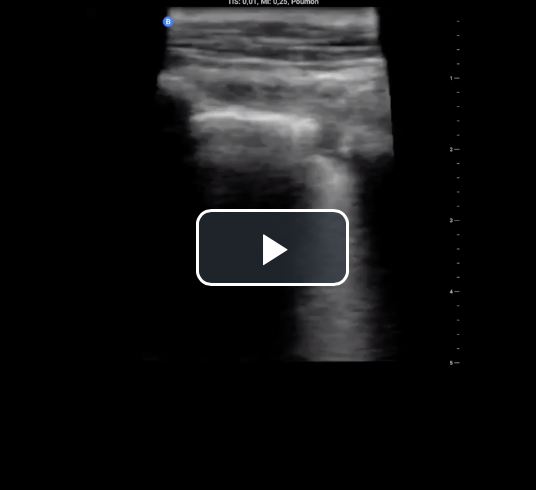 | A. Alveolar syndrome with a small sub-pleural consolidation **(correct)**  B. Interstitial syndrome with >= 3 B-lines  C. Interstitial syndrome with confluent B-line (white lung)  D. Dry lung (A-lines, <3 B-lines, normal lung sliding) |
| Q21. **Visualization of the left lateral quadrant. We observe the spleen, the diaphragm and a large pleural effusion. Click on the spleen.** | 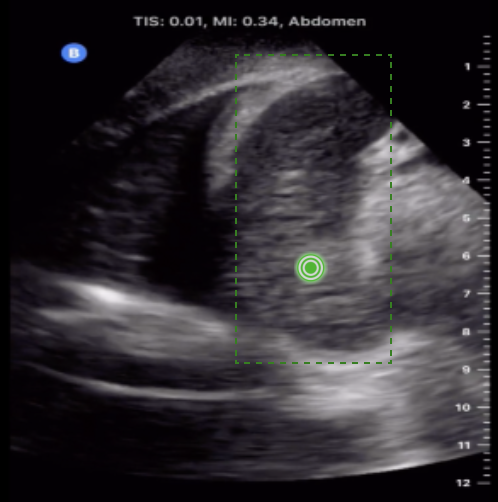 |  |
| Q22. **Which description best matches this video?** | 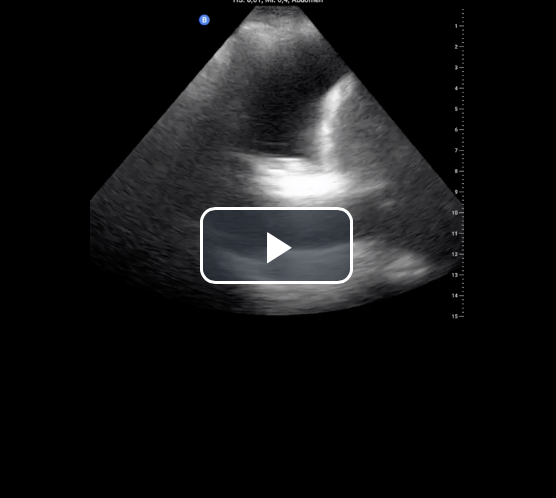 | A. Poor quality image (evaluation of pleural effusion using the "lung" pre-set)  B. Pericardial effusion  C. Rather small quantity pleural effusion, difficult to drain  D. Large pleural effusion, easy to drain **(correct)** |
| Q23. **Which description best matches the image?** | 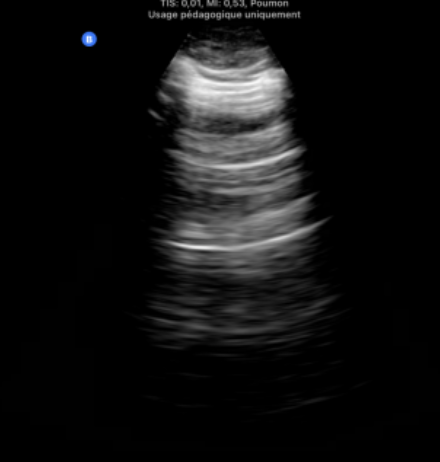 | A. A-lines (dry lung or pneumothorax, impossible to distinguish without video) **(correct)**  B. A-lines (dry lung)  C. A-lines (pneumothorax)  D. A-lines and B-lines |
| Q24. **Click on the sub-pleural consolidation** | 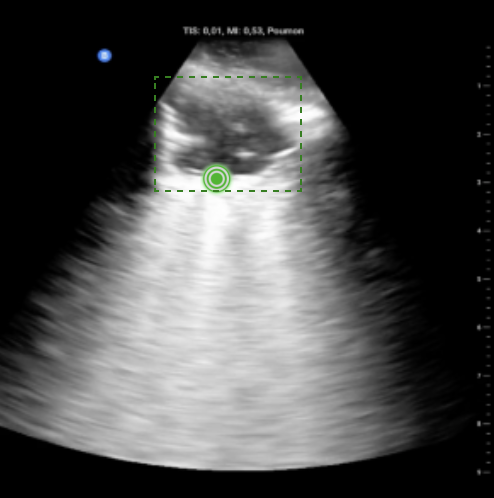 |  |
| Q25. **Which description best matches this image?** | 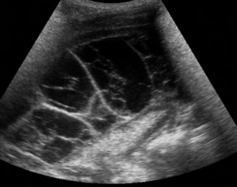 | A. Large complex pleural effusion with plankton sign, difficult to drain  B. Large uncomplicated pleural effusion, easy to drain  C. Smalle pleural effusion, difficult to drain  D. Septated, complex pleural effusion, difficult to drain (**correct)** |
| Q26. **Lung ultrasound is a point-of-care examination. However, it is essential that the results be well documented in the patient's chart. Which statement is correct?** |  | A. All statements are correct **(correct)**  B. It is necessary to document a dated summary in the file: e.g. bilateral interstitial syndrome, large right pleural effusion of approximately 5cm...  C. If printing or saving the images is possible, it is essential to label their anatomic position (e.g. RLQ, LAUQ, RPIQ...) and to note the name of the patient and the date of the examination  D. Correct documentation of results improves patient care and facilitates the work of other colleagues |
| ****Q27.** You examine a 23-year-old patient with cough, dyspnea and fever for 4 weeks.**  **You perform lung ultrasound and find the following images** (cf. video) **in posterior inferior, anterior inferior and lateral right quadrants. How do you proceed?** | 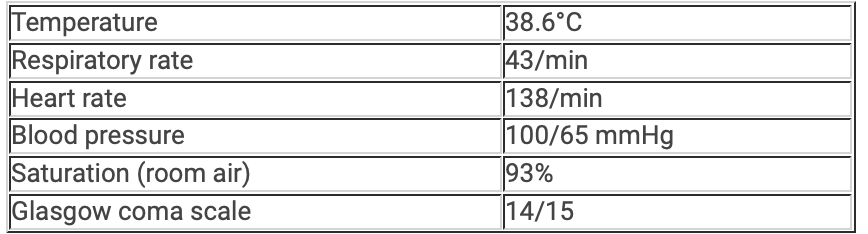  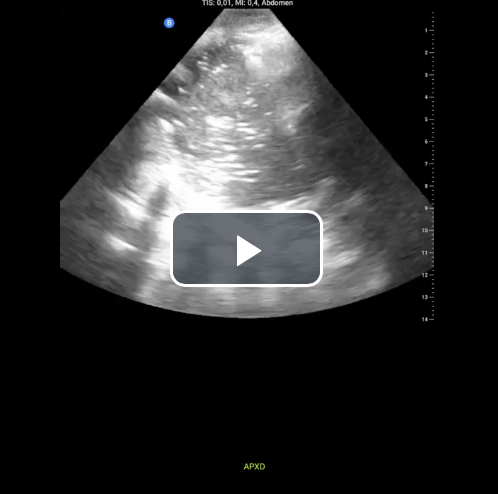 | A. Ultrasound findings confirm a right lobar pneumonia. I start the patient on antibiotics awaiting GenXpert MTB or BK staining results. **(correct)**  B. Ultrasound findings confirm a massive pulmonary embolism. I start the patient on therapeutic dose anticoagulation.  C. Ultrasound findings do not allow to confirm pneumonia. I ask for a chest X-ray before taking any therapeutic decision.  D. Ultrasound findings confirm PCP pneumonia. I start the patient on trimetoprim/sulfamethoxazole and ask for an HIV confirmation test. |
| Q28. **You examine a 55-year old patient known for hypertensive cardiomyopathy. He is consulting for acute dyspnea. You notice bipedal oedema.**  **You perform lung ultrasound and find the following images**(cf. photo)**over all the anterior quadrants . How do you proceed?** | 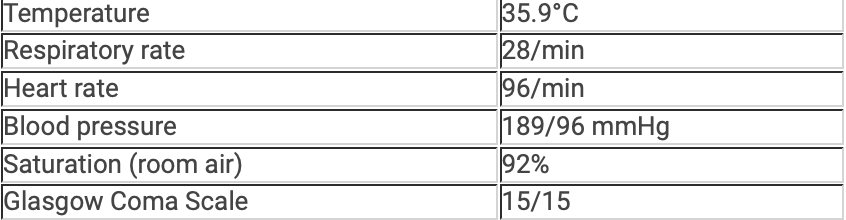  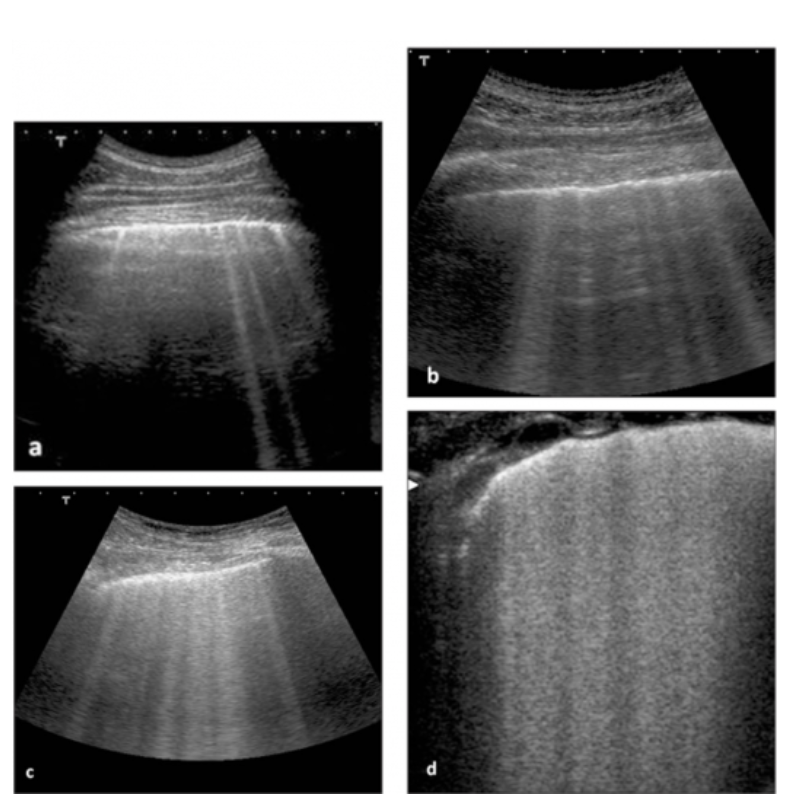 | A. Diffuse interstitial syndrome is suggestive of a fibrotic interstitial lung disease. Refer patient for chest X-ray or CT.  B. Diffuse interstitial syndrome is suggestive of pulmonary embolisms. Obtain D-dimers and perform DVT ultrasound of the lower limbs.  C. Diffuse interstitial syndrome in a high risk cardiovascular patient is suggestive of acute pulmonary edema. Start the patient on diuretics. **(correct)**  D. Diffuse interstitial syndrome is suggestive of viral pneumonia. Ask for a SARS-CoV-2 PCR or antigen test and isolate the patient. Don't start any empiric therapy. |
| Q29. **You examine a 19-year old patient without any history for acute dyspnea and spontaneous chest pain.**  **You perform lung ultrasound and find the following images** (cf. video)**. How do you proceed?** | 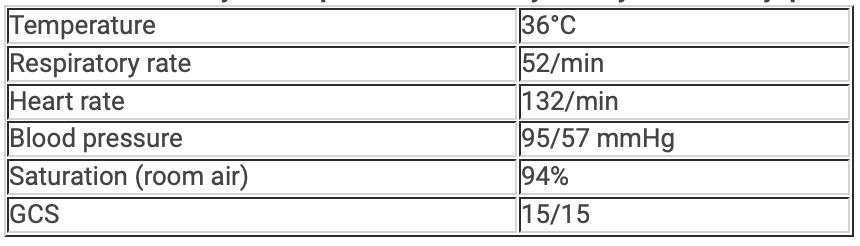  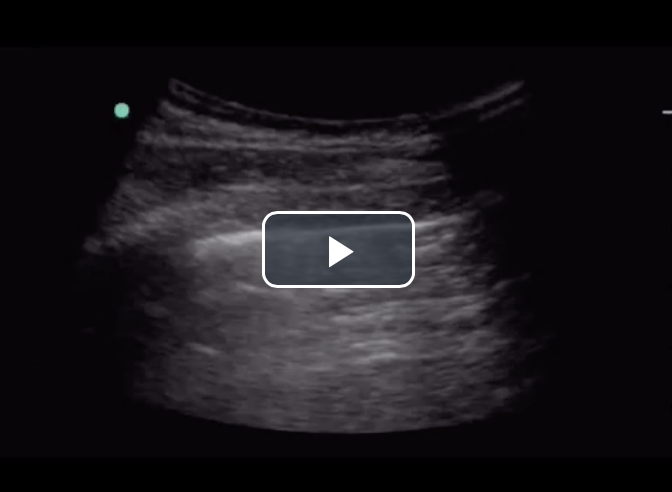 | A. The lung-point identified on ultrasound excludes a pneumothorax and demonstrates normal lung sliding at the costodiaphragmatic recess. The presence of B-lines is rather suggestive of an interstitial syndrome and I continue scanning the other quadrants.  B. The lung-point identified on ultrasound confirms a pneumothorax. I continue scanning to evaluate the extent of the pneumothorax and I prepare for an urgent aspiration. **(correct)**  C. The lung-point identified on ultrasound is suggestive of pneumothorax. It is sufficient to check in M mode that the pleural pulse is present to confirm the diagnosis.  D. The lung-point identified on ultrasound is suggestive of pneumothorax. However, the presence of B-lines could indicate another lung pathology.  A chest X-ray is thus strictly necessary before deciding on any therapeutical intervention. |
| Q30. **You examine a 39-year old patient with cough and dyspnea for at least 2 months. You notice pronounced cachexia.**  **You perform lung ultrasound and find the following image in the right lateral quadrant**(cf photo)**. How do you proceed?** | 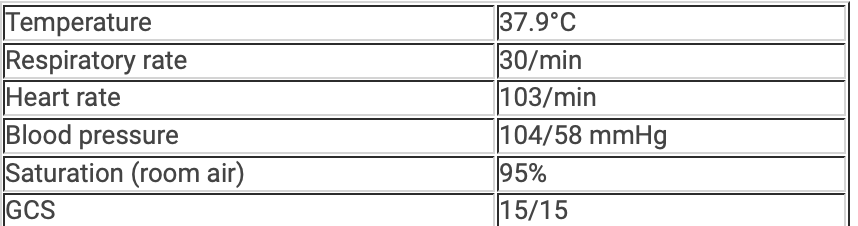 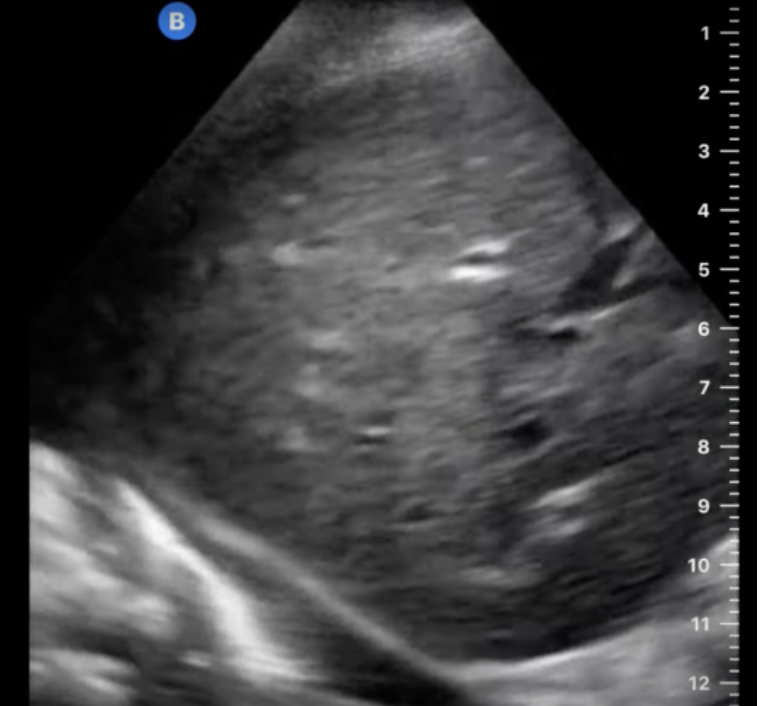 | A. Ultrasound allows to visualize a liver abcess with reactive inflammation around the diaphragm. I refer the patient for puncture/drainage of the liver abcess.  B. Ultrasound confirms the presence of a large uncomplicated pleural effusion with passive atelectasis of the lower right lobe. I prepare a pleural puncture. **(correct)**  C. Ultrasound is suggestive of pleural effusion. However, chest X-ray is strictly necessary to assess whether the effusion is easily drained.  D. Ultrasound is suggestive of empyema. I prepare a pleural puncture and drainage with a large bore canula. |

Table 3: Overview and evaluation of the 5 practical challenges and their respective items.

| Practical Challenges | Evaluated items | | Pass or fail |
| --- | --- | --- | --- |
| P1 : 1 video (5 seconds) of the curtain sign in the right lateral quadrant |  |  | |
|  | Preset | | Pass =1pt /fail = 0pt |
|  | Depth | | Pass =1pt /fail = 0pt |
|  | Gain | | Pass =1pt /fail = 0pt |
|  | saving | | Pass =1pt /fail = 0pt |
|  | Labeling | | Pass =1pt /fail = 0pt |
|  | Interpretability | | Pass =1pt /fail = 0pt |
| P2. 1 video (5 seconds) of the curtains sign in the left lateral quadrant |  | |  |
|  | Preset | | Pass =1pt /fail = 0pt |
|  | Depth | | Pass =1pt /fail = 0pt |
|  | Gain | | Pass =1pt /fail = 0pt |
|  | Saving | | Pass =1pt /fail = 0pt |
|  | Labeling | | Pass =1pt /fail = 0pt |
|  | Interpretability | | Pass =1pt /fail = 0pt |
| P3. 1 photo of the pleural line in M-mode in the right anterior superior quadrant |  | |  |
|  | Preset | | Pass =1pt /fail = 0pt |
|  | Depth | | Pass =1pt /fail = 0pt |
|  | Gain | | Pass =1pt /fail = 0pt |
|  | Saving | | Pass =1pt /fail = 0pt |
|  | Labeling | | Pass =1pt /fail = 0pt |
|  | Interpretability | | Pass =1pt /fail = 0pt |
| P4. 1 video (5 seconds) targeted at 5cm depth on the pleural line in the right anterior superior quadrant |  | |  |
|  | Preset | | Pass =1pt /fail = 0pt |
|  | Depth | | Pass =1pt /fail = 0pt |
|  | Gain | | Pass =1pt /fail = 0pt |
|  | Saving | | Pass =1pt /fail = 0pt |
|  | Labeling | | Pass =1pt /fail = 0pt |
|  | Interpretability | | Pass =1pt /fail = 0pt |
| P5. 1 video (5 seconds) of the pleural line in the left posterior inferior quadrant |  | |  |
|  | Preset | | Pass =1pt /fail = 0pt |
|  | Depth | | Pass =1pt /fail = 0pt |
|  | Gain | | Pass =1pt /fail = 0pt |
|  | Saving | | Pass =1pt /fail = 0pt |
|  | Labeling | | Pass =1pt /fail = 0pt |
|  | Interpretability | | Pass =1pt /fail = 0pt |

Table 4: Link between evaluated items (N=18) and their related electronic quiz questions (1 to 5 per item, refer to Appendix Table 2) and practical challenges results (5 challenges per participant evaluated through 6 items, refer to Appendix Table 3). Evaluation of success using a minimal percent of correct answers or passed challenges per item defined as (n-1)/n with n=the number of questions for 1 item to consider the item as acquired.

| Items | Question (Q) number | N of correct questions needed to passs | %needed to pass |
| --- | --- | --- | --- |
| Indication | Q1, Q2, Q3, Q4 | 3 | 75% |
| Systematic examination | Q6 | 1 | 100% |
| Focused examination | Q7 | 1 | 100% |
| Correct choice of transducer / preset | P1_preset, P2_preset, P3_preset, P4_preset, P5_preset | 4 | 80% |
| Correct depth | P1_depth, P2_depth, P3_depth, P4_depth, P5_depth | 4 | 80% |
| Correct gain | P1_gain, P2_gain, P3_gain, P4_gain, P5_gain | 4 | 80% |
| Saving image | P1_save, P2_save, P3_save, P4_save, P5_save | 4 | 80% |
| Labeling anatomical position | P1_label, P2_label, P3_label, P4_label, P5_label | 4 | 80% |
| Interpretability | P1_interpret, P2_interpret, P3_interpret, P4_interpret, P5_interpret | 4 | 80% |
| Assessment of pleura | Q8, Q11, Q12, Q23 | 3 | 75% |
| Assessment of B-lines | Q9, Q17, Q19 | 2 | 67% |
| Assessment of consolidation | Q15, Q18, Q20, Q24 | 3 | 75% |
| Assessment of pleural effusion | Q13, Q16, Q22 | 2 | 67% |
| Assessment of diaphragm | Q21 | 1 | 100% |
| Assessment of M-mode | Q10, Q14 | 1 | 50% |
| Assessment of whether ultrasoundguided thoracenthesis is safe | Q25 | 1 | 100% |
| Documenting findings in patient chart | Q26 | 1 | 100% |
| Conclusion | Q5, Q27, Q28, Q29, Q30 | 4 | 80% |

Figure 1: training plan (the same for all trainee’s populations)

Link to English lecture slides: [0_Lecture slides EN](https://1drv.ms/f/s!Aj7OkXYo6-Vzr0dUN2W1eCmjw1g1?e=MWTcMb)

Link to French lecture slides: [1_Lecture slides FR](https://1drv.ms/f/s!Aj7OkXYo6-Vzr0bMXxQ7j7kg93Oi?e=ughJXK)


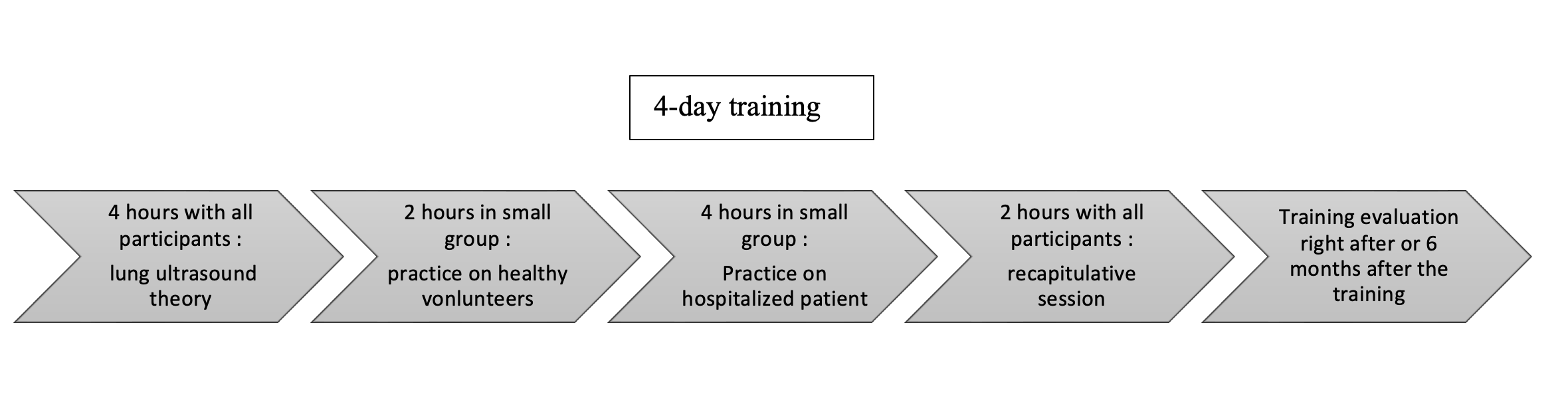

Supplement: Supplementary file 1 — Supplementary Material 1 [file 12909_2024_5925_MOESM1_ESM.docx]
